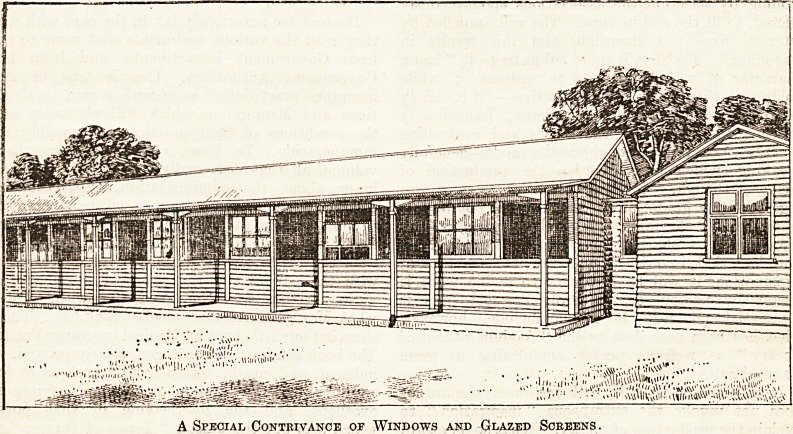# An Open-Air Hospital Ward

**Published:** 1922-11

**Authors:** 


### AN OPEN-AIR HOSPITAL WARD.

SOME years ago Mr. Glendinning Moxham, F.R.I.B.A., was instructed to prepare designs for two open-air wards for the Red Cross Hospital, Danycoed, Swansea, and as the site was in an exposed situation the question of suitable protection had to be considered. The use of canvas blinds with one side practically open was in every way out of the question, as the life of canvas blinds, if used, would be very short and they would not give adequate protection. The wards were therefore arranged to have sliding sashes to the front, and by this means it was possible to have the ward with two-thirds of the front open and also protected by a low-pitched verandah roof. The medical opinion of these wards was very favourable; but the architect came to the conclusion that an improvement could be made on this design, and the result is shown by the accompanying drawing. This design has been most highly spoken of by some of the leading medical men of the day. It provides for the easy opening of the whole of the front portion. The windows, which are juxtaposed, are constructed so that the sashes may be lowered beneath the sill, the sill being fixed at a convenient height, thus permitting the sashes to descend to be flush with the top of the sill, while on the inside the space between the floor and the sill is covered with asbestos slabs, and the outside is enclosed with boarding on battens, thereby providing a boxed-in well for the sashes to descend into when they are opened. It will therefore be seen that a continuous opening can at once be had when desired, the only objects standing in the front being the uprights for carrying the structure as well as the boxed mullions for the sash-weights. The sashes can also be lowered at all times to any desired height, and the contrivance generally has many advantages in this way. Much ingenuity is displayed in the details of this simple and inexpensive design, which is very useful for temporary or permanent hospitals, sanatoria for tuberculosis and open-air schools.

A SPECIAL CONTRIVANCE OF WINDOWS AND GLAZED SCREENS.

## Figures and Tables

**Figure f1:**